# Analysis of Plasminogen Genetic Variants in Multiple Sclerosis Patients

**DOI:** 10.1534/g3.116.030841

**Published:** 2016-05-17

**Authors:** A. Dessa Sadovnick, Anthony L. Traboulsee, Cecily Q. Bernales, Jay P. Ross, Amanda L. Forwell, Irene M. Yee, Lena Guillot-Noel, Bertrand Fontaine, Isabelle Cournu-Rebeix, Antonio Alcina, Maria Fedetz, Guillermo Izquierdo, Fuencisla Matesanz, Kelly Hilven, Bénédicte Dubois, An Goris, Ianire Astobiza, Iraide Alloza, Alfredo Antigüedad, Koen Vandenbroeck, Denis A. Akkad, Orhan Aktas, Paul Blaschke, Mathias Buttmann, Andrew Chan, Joerg T. Epplen, Lisa-Ann Gerdes, Antje Kroner, Christian Kubisch, Tania Kümpfel, Peter Lohse, Peter Rieckmann, Uwe K. Zettl, Frauke Zipp, Lars Bertram, Christina M Lill, Oscar Fernandez, Patricia Urbaneja, Laura Leyva, Jose Carlos Alvarez-Cermeño, Rafael Arroyo, Aroa M. Garagorri, Angel García-Martínez, Luisa M. Villar, Elena Urcelay, Sunny Malhotra, Xavier Montalban, Manuel Comabella, Thomas Berger, Franz Fazekas, Markus Reindl, Mascha C. Schmied, Alexander Zimprich, Carles Vilariño-Güell

**Affiliations:** * Department of Medical Genetics, University of British Columbia, Vancouver, British Columbia, V6T 1Z3, Canada; † Division of Neurology, Faculty of Medicine, University of British Columbia, Vancouver, British Columbia, V6T 1Z3, Canada; ‡ Inserm U 1127, CNRS UMR 7225, Sorbonne Universités, UPMC Univ Paris 06 UMR S 1127, Institut du Cerveau et de la Moelle épinière, ICM, France; § Department of Cell Biology and Immunology, Instituto de Parasitología y Biomedicina López Neyra (IPBLN), CSIC, 18100 Granada, Spain; ** Unidad de Esclerosis Múltiple, Hospital Universitario Virgen Macarena, 41071 Sevilla, Spain; †† Laboratory for Neuroimmunology, Neurosciences, University of Leuven, 1022, Belgium; ‡‡ Department of Neurology, University Hospitals Leuven, University of Leuven, Belgium; §§ Neurogenomiks Group, Universidad del País Vasco (UPV/EHU), 48940 Spain; *** Achucarro Basque Center for Neuroscience, 48170 Spain; ††† IKERBASQUE, Basque Foundation for Science, 48011 Spain; ‡‡‡ Servicio de Neurología, Hospital Universitario Basurto-Osakidetza, 48940 Bilbao, Spain; §§§ Department of Human Genetics, Ruhr University, 44801 Bochum, Germany; **** Department of Neurology, Medical Faculty, Heinrich Heine University, 40225 Düsseldorf, Germany; †††† Department of Neurology, University of Rostock, 18059 Germany; ‡‡‡‡ Department of Neurology, University of Würzburg, 97080 Würzburg, Germany; §§§§ Department of Neurology, University Hospital Bern and University of Bern, 3010 Bern, Switzerland; ***** Institute for Clinical Neuroimmunology, Ludwig Maximilian University, 80539 Munich, Germany; ††††† Department of Neurosurgery, Medical College of Wisconsin, Milwaukee, WI 53226; ‡‡‡‡‡ Institute of Human Genetics, University Medical Center Hamburg-Eppendorf, 20246 Hamburg, Germany; §§§§§ Department of Clinical Chemistry, Ludwig Maximilian University, 80539 Munich, Germany; ****** Institute of Laboratory Medicine and Human Genetics, 78224 Singen, Germany; †††††† Department of Neurology, Sozialstiftung Bamberg Hospital, 96049 Germany; ‡‡‡‡‡‡ Department of Neurology, Focus Program Translational Neuroscience, University Medical Center of the Johannes Gutenberg-University Mainz, 55122 Germany; §§§§§§ Lübeck Interdisciplinary Platform for Genome Analytics, Institutes of Neurogenetics & Integrative and Experimental Genomics, University of Lübeck, 23562, Germany; ******* School of Public Health, Medicine, Imperial College London, SW7 2AZ, UK; ††††††† Department of Neurology, UGC Neurociencias Clínicas. IBIMA-Hospital Regional Universitario de Málaga, 29010 Spain; ‡‡‡‡‡‡‡ Research Laboratory, UGC Neurociencias Clínicas. IBIMA-Hospital Regional Universitario de Málaga, 29010 Spain; §§§§§§§ Immunology and Neurology Service, Multiple Sclerosis Unit, Ramón y Cajal University Hospital-IRYCIS, 28034 Madrid, Spain; ******** Department of Neurology, Instituto de Investigación Sanitaria del Hospital Clínico San Carlos-IdISSC, 28040 Madrid, Spain; †††††††† Department of Immunology, Instituto de Investigación Sanitaria del Hospital Clínico San Carlos-IdISSC, Madrid, Spain; ‡‡‡‡‡‡‡‡ Servei de Neurologia-Neuroimmunologia, Centre d’Esclerosi Múltiple de Catalunya (Cemcat), Institut de Recerca Vall d’Hebron (VHIR), Hospital Universitari Vall d’Hebron, Universitat Autònoma de Barcelona, 08035 Spain; §§§§§§§§ Department of Clinical Neurology, Medical University of Innsbruck, 6020 Austria; ********* Department of Neurology, Medical University of Graz, 8010 Austria; ††††††††† Department of Neurology, Medical University of Vienna, 1090 Austria; ‡‡‡‡‡‡‡‡‡ Institute of Human Genetics, University of Lübeck, 23538 Lübeck, Germany

**Keywords:** multiple sclerosis, genetics, linkage, association, plasminogen

## Abstract

Multiple sclerosis (MS) is a prevalent neurological disease of complex etiology. Here, we describe the characterization of a multi-incident MS family that nominated a rare missense variant (p.G420D) in plasminogen (*PLG*) as a putative genetic risk factor for MS. Genotyping of PLG p.G420D (rs139071351) in 2160 MS patients, and 886 controls from Canada, identified 10 additional probands, two sporadic patients and one control with the variant. Segregation in families harboring the rs139071351 variant, identified p.G420D in 26 out of 30 family members diagnosed with MS, 14 unaffected parents, and 12 out of 30 family members not diagnosed with disease. Despite considerably reduced penetrance, linkage analysis supports cosegregation of PLG p.G420D and disease. Genotyping of PLG p.G420D in 14446 patients, and 8797 controls from Canada, France, Spain, Germany, Belgium, and Austria failed to identify significant association with disease (*P* = 0.117), despite an overall higher prevalence in patients (OR = 1.32; 95% CI = 0.93–1.87). To assess whether additional rare variants have an effect on MS risk, we sequenced *PLG* in 293 probands, and genotyped all rare variants in cases and controls. This analysis identified nine rare missense variants, and although three of them were exclusively observed in MS patients, segregation does not support pathogenicity. *PLG* is a plausible biological candidate for MS owing to its involvement in immune system response, blood-brain barrier permeability, and myelin degradation. Moreover, components of its activation cascade have been shown to present increased activity or expression in MS patients compared to controls; further studies are needed to clarify whether *PLG* is involved in MS susceptibility.

Multiple sclerosis (MS) is a chronic inflammatory demyelinating and neurodegenerative disease of the central nervous system. A genetic contribution to disease susceptibility has been demonstrated in family and twin studies (Ebers *et al.* 1986; Sadovnick 1993; Fagnani *et al.* 2015), and the first pathogenic mutation for MS has been recently identified in *NR1H3* (Wang *et al.* 2016). In addition, a large number genetic risk factors, related primarily to the immune system, have already been identified through association studies (Beecham *et al.* 2013; Sawcer *et al.* 2011). However, with the exception of *HLA-DRB1*, all associated variants have a minor effect on overall disease susceptibility. The identification of genetic components of major effect on disease development is paramount for the generation of physiologically relevant cellular and animal models of human disease, and the generation of treatment strategies that address the underlying biological mechanisms responsible for the onset of MS.

## Materials and Methods

### Participants

A total of 2160 MS patients and 886 unrelated healthy controls from Canada, which includes 1857 multi-incident families, collected through the Canadian Collaborative Project on the Genetic Susceptibility to Multiple Sclerosis (CCPGSMS), were included in this study (Sadovnick *et al.* 1998). Five independent European cohorts consisting of 2391 MS patients and 672 healthy controls from France, 4288 patients and 4018 controls from Spain, 3733 patients and 2722 controls from Germany, 1006 patients and 504 controls from Belgium, and 925 patients from Austria, were used for replication. All patients were diagnosed with MS according to published criteria (Poser *et al.* 1983; McDonald *et al.* 2001; Polman *et al.* 2005), and the demographics for each cohort are presented in Table 1. The ethical review board at each institution approved the study, and all participants provided written informed consent.

**Table 1:** Logistic regression analysis for PLG p.G420D (rs139071351) and risk of MS

	Group	Gender M(%)	Age (mean ± SD)	Age at onset (mean ± SD)	Genotypes (GA/GG)	P-Value	OR (95% CI)
Canada	Controls	51.0	67.1 ± 9.8		1/880	0.046	10.19 (1.04–267.89)
	MS patients	26.9	46.7 ± 11.7	31.0 ± 9.7	12/2091		
France	Controls	39.1	39.3 ± 13.1		4/668	0.049	2.69 (1.00–9.37)
	MS patients	30.0	49.1 ± 11.4	30.5 ± 9.7	32/2359		
Spain	Controls	40.5	42.8 ± 12.8		34/3984	0.475	1.20 (0.73–1.96)
	MS patients	34.8	44.5 ± 11.5	30.9 ± 9.8	42/4246		
Germany	Controls	40.3	41.3 ± 16.8		11/2711	0.476	1.31 (0.63–2.84)
	MS patients	29.2	40.5 ± 11.3	30.8 ± 10.3	21/3712		
Belgium	Controls	47.2	56.2 ± 14.7		5/499	0.747	0.81 (0.23–3.04)
	MS patients	34.0	48.3 ± 13.1	33.3 ± 10.9	6/1000		
Austria	MS patients	29.8	49.2 ± 12.1	28.7 ± 9.1	7/918	NA	NA
Combined	Controls	41.8	44.3 ± 15.9		55/8742	0.117	1.32 (0.93–1.87)
	MS patients	31.0	45.1 ± 12.1	30.9 ± 9.9	120/14326		

M, male; OR, odds ratio; CI, confidence interval; NA, not applicable.

### Exome sequencing

**Figure 1:**
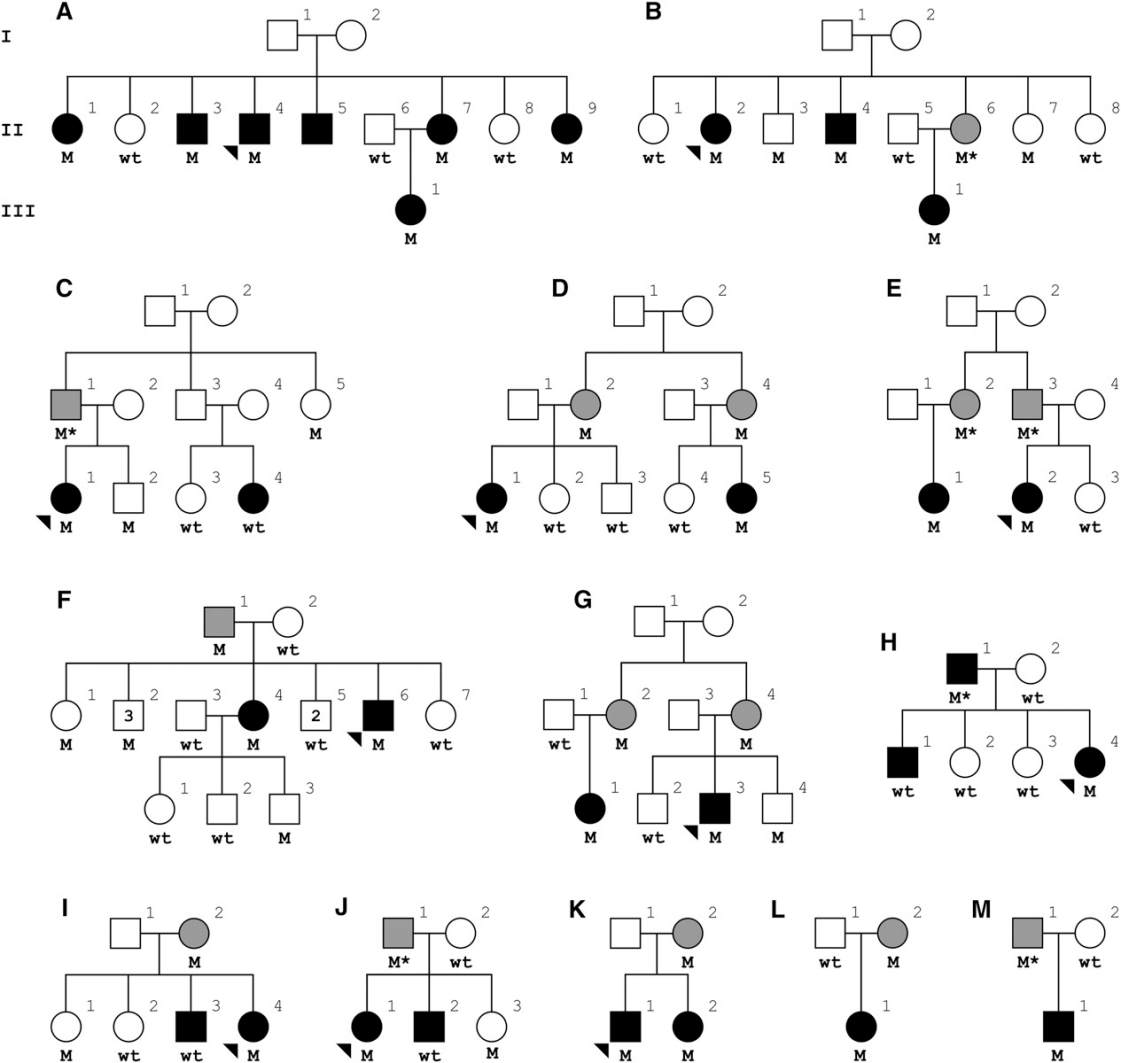
Simplified pedigrees for families presenting the PLG p.G420D variant. Males are represented by squares and females by circles, the proband is indicated with an arrow head. Patients diagnosed with MS have black filled symbols, and carriers of unknown clinical phenotype have gray filled symbols. Heterozygote carriers (M) and wild-type (wt) genotypes are indicated. An asterisk indicates an inferred carrier. Pedigree A was used for exome analysis, and, with the exception of pedigree E, which is of Asian descent, all families are of Caucasian ancestry.

We performed exome sequencing in three patients diagnosed with MS (pedigree A; II-1, II-4, and III-1) from a multi-incident family (Figure 1). Exonic regions were enriched using an Ion AmpliSeq exome kit (57.7 Mb), and sequenced in an Ion Proton sequencer (Life Technologies, Carlsbad, CA) with a minimum average coverage of 50 reads per base, and an average read length of 150 bases. The Ion Torrent Server v4 was used to map reads to NCBI Build 37.1 reference genome using the Torrent Mapping Alignment Program (TMAP), and to identify variants differing from the reference. Sequences with a mapping Phred quality score under 20, fewer than five reads, or over 95% strand bias were excluded from further analysis.

### Sequencing, genotyping, and statistical analysis

Sanger sequencing was used to genotype amplicons containing exome variants of interest, and all 19 coding exons, and exon–intron boundaries, of plasminogen (*PLG*, NM_000301.3) by polymerase chain reaction (PCR) as previously described (Sadovnick *et al.* 2013). Nine tagging SNPs (tSNPs) spanning a 61 kb region encompassing the *PLG* locus were selected based on HapMap data (version 3, release 27) using Haploview software (Barrett *et al.* 2005). Selected tSNPs captured over 92% of the polymorphic variation in the region [minor allele frequency (MAF) > 5%, and *r*^2^ > 0.8] in Caucasian population standards. Genotyping of variants was performed using a combination of TaqMan probes and Sequenom MassArray iPLEX as previously described (Traboulsee *et al.* 2014; Nishioka *et al.* 2010). Genotyping success rate was over 99.4% for all variants, and without deviation from Hardy-Weinberg equilibrium expectation (p-value > 0.005). Statistical association was determined using logistic regression analysis adjusted for age and gender, in addition, the combined cohort analysis was adjusted for site. Genotypes were dichotomized as presence *vs.* absence of the minor allele (dominant model). The combined dataset was obtained by pooling samples from all populations. Segregation was quantified using nonparametric and parametric linkage analysis. Nonparametric linkage analysis was performed using SimWalk2 software (version 2.91), and NPL-All statistic (Sobel *et al.* 2001). Two-point parametric logarithm of odds (LOD) scores were obtained with MLINK, assuming a dominant model, with a fully penetrant disease, and without phenocopies (Ott 1989). All MS patients were treated as affected, noncarrier individuals as healthy, and unaffected mutation carriers were treated as having an unknown disease status. The deleterious allele was defined with a 0.0001 frequency, and the marker-allele frequency was determined empirically from genotyped individuals.

### Haplotype analysis

Microsatellite markers spanning the *PLG* locus between D6S1633 and D6S297 were chosen to define the disease-carrying haplotype (Supplemental Material, Table S1). All family members from those families identified with the PLG p.G420D mutation were genotyped. One primer for each pair was labeled with a fluorescent tag, and PCR reactions were performed under standard conditions. PCR products were run on an ABI 3730xl (Life Technologies, Carlsbad, CA), and analyzed using GeneMapper 4.0. Marker sizes were normalized to those reported in the CEPH database and manually phased within each family.

### Data availability

The authors state that all data necessary for confirming the conclusions presented in the article are represented fully within the article.

## Results

To identify genes and variants of major effect on MS susceptibility, we applied exome sequencing analysis to a multi-incident family consisting of 12 individuals over three generations, with DNA available for nine family members, including six diagnosed with MS (Figure 1A). Exome analysis of II-1, II-4, and III-1, identified 47479, 46545, and 46580 variants, respectively. Of those, 25 missense variants with a MAF below 1% from public and proprietary databases of variants were identified in all three individuals (Table S2). Segregation in additional family members identified 10 variants shared among at least five of the six family members diagnosed with MS for whom DNA was available, and no more than one of the two unaffected blood relatives. Three of these variants were subsequently excluded as they were identified at a frequency over 1% in 366 ethnically matched controls (Table S2). The seven remaining variants were genotyped in a multi-ethnic cohort consisting of 2160 MS patients and 886 unrelated healthy controls from Canada. Three variants [TGFBI, p.V608L (ss1467426521); SPINK13, p.C72R (ss1467426567); OR1E1, p.D96Y (ss1467426912)] appear to be private as they were not observed in any of the other samples genotyped in this study, and have not been described in public databases of variants (Abecasis *et al.* 2012; Exome Aggregation Consortium *et al.* 2015). ARHGAP10, p.T518K (rs375188932), with a reported MAF of 5 × 10^−5^ in the ExAC database, was also not observed in any additional samples. Segregation of these four variants within the exome sequenced family is provided in Figure S1. Of the remainder, SPATA18 p.P286L (rs150116592) was identified in two MS patients, UNC45B p.R776Q (rs34242925) was identified in one patient and one control, and PLG p.G420D (rs139071351) in 12 MS patients and one control.

**Figure 2:**
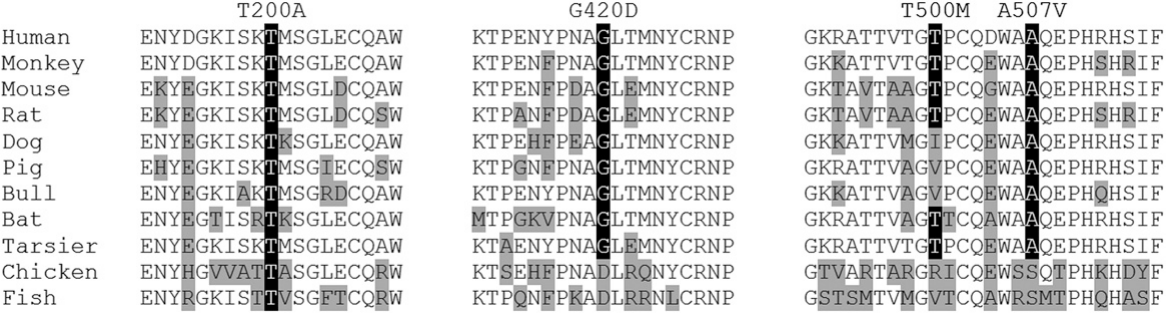
PLG variants and cross-species conservation. Protein orthologs were aligned via ClustalO. Amino acid positions for PLG variants are highlighted in black. Protein orthologs with amino acid positions differing from those of the human sequence are indicated in gray. RefSeq accession numbers: *Homo sapiens* NP_000292.1, *Macaca mulatta* NP_001036540.1, *Mus musculus* NP_032903.3, *Rattus norvegicus* NP_445943.1, *Canis lupus familiaris* NP_001273889.1, *Sus scrofa* NP_001038055.1, *Bos taurus* NP_776376.1, *Myotis davidii* ELK34830.1, *Tarsius syrichta* XP_008066085.1, *Gallus gallus* XP_419618.2, and *Danio rerio* AAH59801.1.

Segregation for variants identified in *SPATA18* and *UNC45B* did not support cosegregation with disease in additional families, and were excluded from further analysis (Figure S1). Segregation of PLG p.G420D identified the variant in 26 out of 30 family members diagnosed with MS (87%), 14 parents of MS patients (including eight obligate carriers) not known to suffer from MS, and 12 out of 30 family members not diagnosed with disease (Figure 1, B–M). To quantifiably assess segregation, we performed nonparametric and parametric linkage analysis for PLG p.G420D. The more conservative nonparametric score resulted in a LOD score of 1.29, whereas parametric linkage analysis resulted in a maximum LOD score of 5.48 (θ = 0.05), despite a penetrance estimate of 50%. Additional support for a role in disease susceptibility is provided by the level of conservation for the glycine residue in mammals, indicating the importance of this amino acid for protein function (Figure 2). Haplotype analysis of PLG p.G420D carriers between D6S1633 and D6S297 did not identify a shared haplotype among families (Table S1), thus suggesting that PLG p.G420D is a mutational hotspot that has independently arisen in each family rather than being inherited from a common ancestor.

Clinical details were available for 17 PLG p.G420D carriers, five males and 12 females (Table S3). The disease course observed in these carriers was predominantly consistent with relapsing-remitting MS, or secondary progressive MS, with only two patients presenting primary progressive MS. On average, the age at onset of disease was 35.1 years (SD ± 9.1), with a disease duration of 19.9 years (SD ± 10.4). Disease severity was overall relatively moderate, with an average expanded disability status scale (EDSS) score of 3.92 (SD ± 2.9) and a median of 2.75.

Association analysis of PLG p.G420D was performed in Caucasian samples from Canada already genotyped for the identification of additional PLG p.G420D families. This subset consists of 2103 MS patients and 881 controls, and resulted in a marginally significant association with disease risk (*P* = 0.046), and an odds ratio (OR) of 10.19 (Table 1). In order to validate this association we genotyped PLG p.G420D in five independent cohorts from Europe consisting of 12343 MS patients and 7916 healthy controls. Logistic regression analysis corrected for age and gender identified a similarly marginal association with disease in the French cohort (*P* = 0.049; OR = 2.69), whereas no association was observed for any additional cohort (Table 1). Although the combined dataset did not result in a significant association with disease risk (*P* = 0.117), with the exception of Belgium which is the smallest set, all cohorts resulted in OR greater than 1, indicating a higher prevalence of PLG p.G420D in MS patients than controls.

To assess whether common variants in *PLG* lead to an increased susceptibility to develop MS, we identified nine tSNPs spanning the entire *PLG* loci, and genotyped them in 2103 MS patients and 881 controls from Canada (Table S4). Association analysis failed to identify a significant association between any of the tSNPs and susceptibility to MS (*P* > 0.05). Since common variants in *PLG* do not appear to have an effect on MS disease risk, we assessed for the presence of additional rare PLG substitutions in MS patients. To this end, we sequenced all *PLG*-coding exons in 293 familial probands from Canada, which identified 11 silent and 11 missense variants (Supplementary Table S5). Of those, nine missense variants with a MAF below 1% in at least two of three publicly available databases (1000G, ExAC, or ESP) were genotyped in cases and controls from Canada (Abecasis *et al.* 2012; Exome Aggregation Consortium *et al.* 2015; Exome Sequencing Project 2014). This analysis identified six variants (p.K38E, p.R89K, p.R261H, p.R490Q, p.A494V, and p.R523W) at similar frequencies in MS patients and controls; whereas p.T200A (rs149145958), p.T500M (rs140970354) and p.A507V (rs372603134) were identified only in eight, two and one MS patient, respectively (Table 2). Despite all three variants being predicted likely damaging to protein function with a phred-scaled CADD score of 29.3, 14.4, and 18.9 for p.T200A, p.T500M, and p.A507V, respectively (Kircher *et al.* 2014), and two of them being evolutionarily conserved (Figure 2), segregation and parametric linkage analysis, which resulted in negative LOD scores, does not support a role for these variants in disease pathogenicity (Figure S2).

**Table 2:** Case-control frequency for rare missense PLG variants identified in MS patients

dbSNP ID*^a^*	Chromosome and Position	Nucleotide Change	Protein Change	Minor Allele Frequency		
				ExAC*b*	Controls (*n*)	MS (*n*)
rs73015965	6:161127501	A/G	p.K38E	0.003	0.006 (10)	0.007 (28)
rs143079629	6:161128812	G/A	p.R89K	0.007	0.010 (16)	0.010 (44)
rs149145958	6:161135876	A/G	p.T200A	0.001	0	0.002 (8)
rs4252187	6:161137790	G/A	p.R261H	0.003	0.007 (12)	0.005 (24)
rs140537724	6:161152807	G/A	p.R490Q	0.001	0.002 (3)	0.002 (9)
rs4252128	6:161152819	C/A	p.A494V	0.008	0.005 (8)	0.005 (20)
rs140970354	6:161152837	C/T	p.T500M	0.0002	0	0.0005 (2)
rs372603134	6:161152858	C/T	p.A507V	0.0001	0	0.0002 (1)
rs4252129	6:161152905	C/T	p.R523W	0.007	0.012 (19)	0.013 (56)

a dbSNP Build 138.

b The Exome Aggregation Consortium (ExAC) database.

## Discussion

Exome sequencing analysis in a multi-incident family suffering from MS has nominated PLG p.G420D as a putative new risk factor for MS. Although four private missense variants cannot be conclusively excluded as a potential cause of disease in this kindred, and copy number changes were not evaluated, the identification of PLG p.G420D in 12 additional MS patients, and one control from Canada, suggests a role for *PLG* in MS susceptibility. Genotyping of additional family members from multi-incident families with PLG p.G420D resulted in positive cosegregation of the variant and disease, albeit with 50% reduced penetrance (Figure 1). Additional support for pathogenicity was sought from a large case-control cohort of MS patients from Europe, and, although most populations present a higher prevalence of PLG p.G420D in MS patients than controls, a nominally significant difference was observed only in the French cohort (Table 1). A possible Acadian origin of PLG p.G420D was considered due to the marginal associations in the French and Canadian population; however, the wide geographical distribution of variant carriers from Canada, and the lack of a shared ancestral haplotype (Table S1), do not support this hypothesis. Association analysis for PLG p.G420D in the entire cohort resulted in a nonsignificant p-value of 0.117, and an OR of 1.32. Despite the overall lack of association observed, it is possible that carriers of the PLG p.G420D variant have an increased risk of developing MS, as suggested by the OR and initially observed familial segregation pattern. In contrast, common *PLG* tagging variants genotyped in this study were clearly not associated with MS risk in the Canadian population (Table S4). This data corroborates previously described genome wide association studies that did not nominate common variants in *PLG* as a risk factor for MS (Beecham *et al.* 2013; Sawcer *et al.* 2011).

Sequencing of *PLG* in MS patients from Canada led to the identification of nine rare missense variants (Table 2). Six of these were subsequently identified at a similar frequency in MS patients and controls, suggesting they are not likely to have an effect on MS risk. Interestingly one of these variants (p.K38E, rs73015965) has been described as the cause of PLG deficiency type I when identified in homozygous or compound heterozygous form (Tefs *et al.* 2006). Similarly, p.R523W (rs4252129) has been associated with decreased plasma PLG levels (Ma *et al.* 2014). Severe PLG deficiency type I has been causally linked to ligneous conjunctivitis, a rare chronic inflammatory disease of mainly mucous membranes. Although there is no indication that heterozygous carriers are at an increased risk of developing disease (Tefs *et al.* 2006), *PLG* dysregulation could lead to an increased susceptibility to inflammatory and autoimmune diseases. In our study, three additional variants (p.T200A, p.T500M, and p.A507V) not known to cause hypoplasminogenemia, were observed exclusively in MS patients. Although the allelic frequencies and segregation for rare missense PLG variants do not initially support a role in disease susceptibility, genotyping in additional MS patients is warranted to fully define these preliminary findings. PLG p.T200A seems of particular interest, as it was identified in eight MS patients and no controls (Table 2), it is evolutionary conserved (Figure 2), and a threonine to proline substitution at the same position has been identified in a patient with severe type I PLG deficiency (Tefs *et al.* 2006).

*PLG* is a plausible biological candidate for MS susceptibility as it is involved in the inflammatory response, blood-brain barrier (BBB) permeability, neuronal viability, and myelin degradation (Syrovets *et al.* 2012; Yao and Tsirka 2011; Chen and Strickland 1997; Cuzner and Opdenakker 1999). PLG has been shown to play a role in the immune response, with plasmin deficiency, the active form of PLG, resulting in a compromised inflammatory response in mouse brain (Hultman *et al.* 2014). Microglia and astrocytes are the primary mediators of inflammation in the central nervous system, and fibrin has been shown to activate their immune response by stimulating the production of inflammatory mediators, including proinflammatory cytokines and reactive oxygen species, as well as act as a chemoattractant for immune cells (Syrovets *et al.* 2012; Hultman *et al.* 2014).

Genetic variants in *PLG* may also have an effect on brain inflammation by altering the BBB permeability. Plasmin alters BBB permeability by inducing morphological changes in brain astrocytes and endothelial cells through the reorganization of the actin cytoskeleton and the redistribution of tight junction proteins (Niego and Medcalf 2014; Yao and Tsirka 2011). In addition to its effects on the inflammatory response and BBB permeability, plasmin has also been shown to affect neuronal viability, including sprouting, plasticity, and extracellular matrix-related neuronal death (Chen and Strickland 1997; Nakagami *et al.* 2000; Wu *et al.* 2000).

Plasmin activates highly active matrix metalloproteinases (MMPs) which are recognized as key proteases in the demyelination process. Synthetic inhibitors of MMPs have been found to ameliorate clinical symptoms and pathological signs in experimental autoimmune encephalomyelitis (EAE) animal models (Cuzner and Opdenakker 1999); minocycline, which has several immunomodulating activities including the inhibition of MMP-9, has been used successfully in clinical trials as an add-on therapy for MS patients (Metz *et al.* 2009).

Despite the existence of extended families with a high incidence of MS (Fagnani *et al.* 2015; Sadovnick 1993), only one rare pathogenic mutations has been reported (Wang *et al.* 2016). In this study, the implementation of exome sequencing analysis in a multi-incident MS family nominated PLG p.G420D as a potential susceptibility risk for MS. Additional support was provided by 10 additional multi-incident MS families in which the variant segregates with disease, albeit with reduced penetrance. Disappointingly, genotyping of PLG p.G420D in a large European case-control cohort failed to identify a significant association with MS, thus not supporting a role for PLG p.G420D in disease. Despite this lack of association, dysregulation of the PLG/plasmin activation cascade is a plausible pathomechanism of MS, which, in conjunction with the positive segregation of PLG p.G420D in families (Figure 1), the overall higher incidence of PLG p.G420D carriers in European MS patients (Table 1), and the identification of additional rare PLG substitutions in MS patients not observed in controls (Table 2), warrants further genetic and functional characterization of *PLG* in order to elucidate its potential role on MS susceptibility and pathogenesis.

## Supplementary Material

Supplemental Material
