# Supplementary material for: Analysis of Plasminogen Genetic Variants in Multiple Sclerosis Patients
Source: G3 (Bethesda). 2016 May 17;6(7):2073–9. doi: 10.1534/g3.116.030841 (PMC4938660; doi:10.1534/g3.116.030841)
Supplement: Supplemental Material [file supp_6_7_2073__index.html]

Analysis of Plasminogen Genetic Variants in Multiple Sclerosis Patients — Supplemental Material 

# Analysis of Plasminogen Genetic Variants in Multiple Sclerosis Patients

## Supplemental Material for Sadovnick *et al.*, 2016

**Files in this Data Supplement:**

- Figure S1 - Segregation analysis of exome variants. (.pdf, 70 KB)
- Figure S2 - Segregation analysis of PLG variants not observed in controls. (.pdf, 75 KB)
- Table S1 - Chromosome 6q25.3-27 haplotypes carrying PLG p.G420D. (.pdf, 46 KB)
- Table S2 - Novel and rare (MAF<0.01) missense variants shared by family A members II-1, II-4 and III-1. (.pdf, 53 KB)
- Table S3 - Clinical features for PLG p.G420D carriers. (.pdf, 46 KB)
- Table S4 - Logistic regression analysis for PLG-tagging SNPs. (.pdf, 43 KB)
- Table S5 - PLG-coding variants identified in MS patients. (.pdf, 50 KB)
