# Supplementary material for: Analysis of Plasminogen Genetic Variants in Multiple Sclerosis Patients
Source: G3 (Bethesda). 2016 May 17;6(7):2073–9. doi: 10.1534/g3.116.030841 (PMC4938660; doi:10.1534/g3.116.030841)

Figure S1. Segregation analysis of exome variants. Males are represented by squares and females by circles, with the proband indicated with an arrow head. Patients diagnosed with MS have black filled symbols. Heterozygote carriers (M) and wild-type (WT) genotypes are indicated.

ARHGAP10 p.T518K (rs375188932)

Original family (Fig1 A)

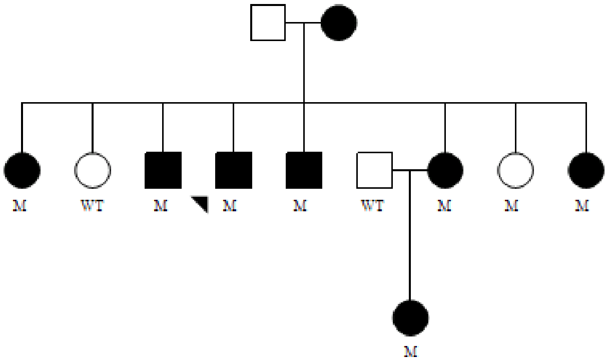

TGFBI p.V608L (ss1467426521)

Original family (Fig1 A)

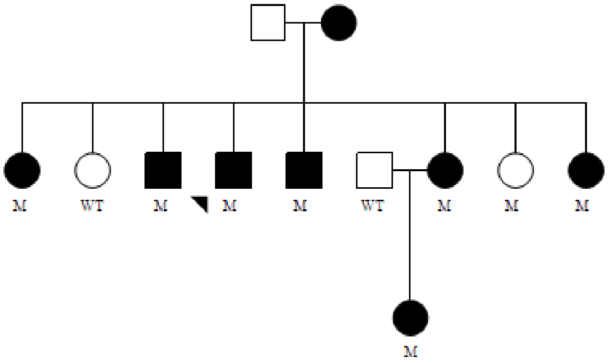

SPINK13 p.C72R (ss1467426567)

Original family (Fig1 A)

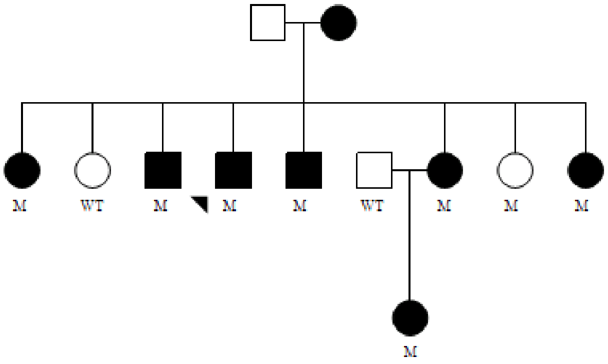

OR1E1 p.D96Y (ss1467426912)

Original family (Fig1 A)

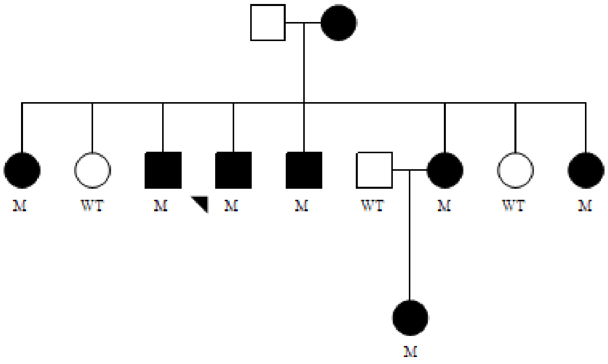

SPATA18 p.P286L (rs150116592)

Original family (Fig1 A)

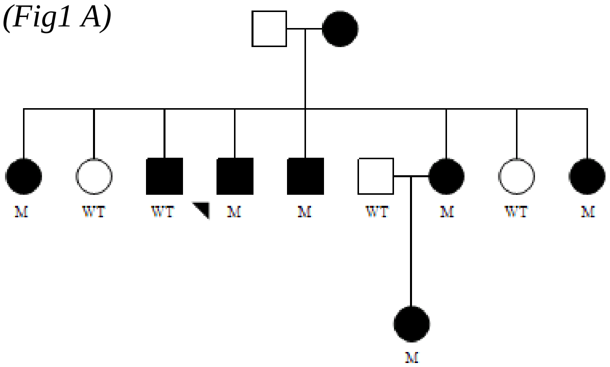

Additional families

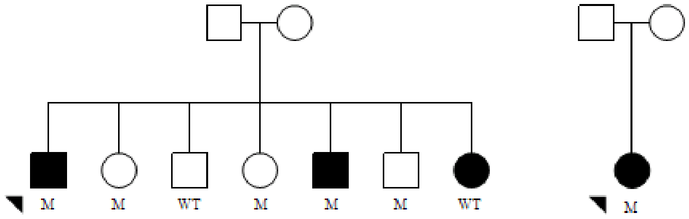

UNC45B p.R776Q (rs34242925)

Original family (Fig1 A)

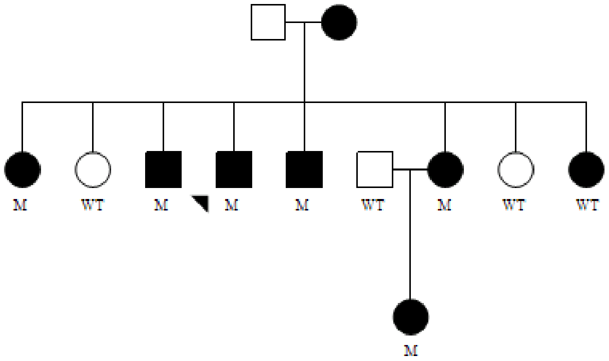

Additional family

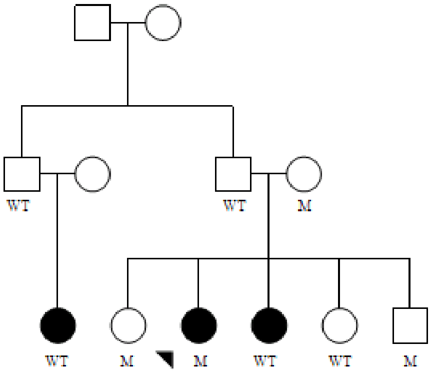

Supplement: Supplemental Material [file supp_g3.116.030841_FigureS1.pdf]
