# Supplementary material for: Analysis of Plasminogen Genetic Variants in Multiple Sclerosis Patients
Source: G3 (Bethesda). 2016 May 17;6(7):2073–9. doi: 10.1534/g3.116.030841 (PMC4938660; doi:10.1534/g3.116.030841)
Supplement: Supplemental Material [file supp_g3.116.030841_TableS1.pdf]

Table S1. Chromosome 6q25.3-27 haplotypes carrying PLG p.G420D. Microsatellite allele sizes are given in base pairs consistent with Centre d'Etude du Polymorphisme Humain (CEPH) standards. For markers with an unknown phase both alleles are given. Markers are shown with their physical locations according to NCBI Build 37.1.

**Marker**	**Position**	**Family ID**												
		**A**	**B**	**C**	**D**	**E**	**F**	**G**	**H**	**J**	**I**	**K**	**L**	**M**
D6S1633	157,016,443	129	125	121	129	119	131	117	121	117	121	121/129	127	121/129
D6S415	157,794,580	263	275	275	265	263	261	261	265	261	265	267	261	263/275
D6S1655	158,300,596	149	149	149/163	165	151	155	149	155	153	155	149	155	149
D6S437	158,683,884	135	153	131	131	161	163	131	147	131	135	131/153	131	131/159
D6S1581	160,196,757	219	229	219	219	219	219	219	219	227	219	219/227	219	215
rs139071351	161,152,085	A	A	A	A	A	A	A	A	A	A	A	A	A
D6S305	162,115,166	218	204	226	224	218	204	218	218	232	204	226	204	226
D6S1599	162,759,584	151	147	131	131	131	133	133	133	143	133	133	133	155
D6S1277	164,217,713	290	296	294/296	296	296	298	302	298	298	298	298	298	298
D6S1719	165,989,655	180	184	182	178	178	180	174	174	182	178	178	178	182
D6S297	167,156,674	222	212	222	214	212	222	212	212	212	222	222	212	212
