# Supplementary material for: Analysis of Plasminogen Genetic Variants in Multiple Sclerosis Patients
Source: G3 (Bethesda). 2016 May 17;6(7):2073–9. doi: 10.1534/g3.116.030841 (PMC4938660; doi:10.1534/g3.116.030841)
Supplement: Supplemental Material [file supp_g3.116.030841_TableS2.pdf]

Table S2. Novel and rare (MAF<0.01) missense variants shared by family A members II-1, II-4 and III-1. Chromosomal positions are provided in reference to NCBI Build 37.1.

Chr	Position	Gene	nucleotide change	Transcript	AA change	dbSNP rs/ss number	Selection/exclusion criteria
1	865,665	SAMD11	G/A	NM_152486	R68Q	rs145442390	Does not segregate with disease
1	1,961,467	GABRD	G/A	NM_000815	G369S	rs199865640	Does not segregate with disease
1	226,573,339	PARP1	G/A	NM_001618	L293F	rs149619679	Does not segregate with disease
1	228,505,276	OBSCN	G/T	NM_001098623	R4558L	rs199865640	Does not segregate with disease
1	230,921,738	CAPN9	G/A	NM_016452	G472E	ss1467426444	Does not segregate with disease
4	52,943,043	SPATA18	C/T	NM_145263	P286L	rs150116592	MS frequency = 0.001<br>Control frequency = 0
4	148,886,277	ARHGAP10	C/A	NM_024605	T518K	rs375188932	Private variant
4	155,254,540	DCHS2	G/C	NM_001142552	H940Q	rs79215995	Control frequency = 0.01
5	40,852,774	CARD6	T/C	NM_032587	L447P	rs143022216	Does not segregate with disease
5	57,790,693	GAPT	A/T	NM_152687	E110D	rs147191680	Does not segregate with disease
5	94,927,243	ARSK	C/T	NM_198150	P337L	rs149766065	Does not segregate with disease
5	98,224,802	CHD1	T/A	NM_001270	Y774F	rs144567251	Does not segregate with disease

5	123,980,164	ZNF608	T/C	NM_020747	K1299R	rs113873110	Control frequency = 0.03
5	135,396,541	TGFBI	G/C	NM_000358	V608L	ss1467426521	Private variant
5	147,661,772	SPINK13	T/C	NM_001040129	C72R	ss1467426567	Private variant
5	176,798,224	RGS14	G/A	NM_006480	R438K	ss1467426609	Does not segregate with disease
6	161,152,085	PLG	G/A	NM_000301	G420D	rs139071351	MS frequency = 0.005 Control frequency = 0.001
10	99,240,767	MMS19	C/T	NM_022362	R65Q	ss1467426865	Does not segregate with disease
15	42,185,151	SPTBN5	G/A	NM_016642	R74C	rs62002144	Does not segregate with disease
15	44,865,000	SPG11	T/C	NM_001160227	N1962S	rs140824939	Does not segregate with disease
16	66,804,108	CCDC79	G/T	NM_001136505	S459R	rs189708354	Does not segregate with disease
17	3,301,419	OR1E1	C/A	NM_003553	D96Y	ss1467426912	Private variant
17	33,507,649	UNC45B	G/A	NM_001033576	R776Q	rs34242925	MS frequency = 0.0005 Control frequency = 0.001
20	68,396	DEFB125	C/T	NM_153325	R16W	rs138777928	Control frequency = 0.01
22	46,780,446	CELSR1	C/T	NM_014246	E2293K	rs140996267	Does not segregate with disease
