# Supplementary material for: Analysis of Plasminogen Genetic Variants in Multiple Sclerosis Patients
Source: G3 (Bethesda). 2016 May 17;6(7):2073–9. doi: 10.1534/g3.116.030841 (PMC4938660; doi:10.1534/g3.116.030841)
Supplement: Supplemental Material [file supp_g3.116.030841_TableS3.pdf]

Table S3 Clinical features for PLG p.G420D carriers. RR, Relapsing-remitting MS; PP, primary progressive MS; SP, secondary progressive MS; EDSS, expanded disability status scale; NA, not available.

Pedigree	Individual	Gender	Disease course	Age	Age at onset	Disease duration	EDSS score
A	II-1	F	RR	69	41	28	-
A	II-3	M	PP	65	26	39	-
A	II-4	M	RR	63	44	19	2
A	II-7	F	RR	58	20	38	-
A	II-9	F	RR	52	23	29	-
B	II-2	F	RR	59	35	24	3.5
C	III-1	F	RR	52	37	15	3
D	III-1	F	RR	55	36	19	2
E	III-2	F	SP	53	26	27	8
F	II-6	M	RR	66	54	12	6.5
G	III-1	F	PP	53	45	8	-
G	III-3	M	RR	41	27	14	1
H	II-4	F	RR	50	44	6	1
I	II-4	F	SP	43	31	12	7.5
J	II-1	F	RR	59	37	22	8
L	II-1	F	RR	43	40	3	2.5
M	II-1	M	-	53	30	23	2
