# Supplementary material for: Analysis of Plasminogen Genetic Variants in Multiple Sclerosis Patients
Source: G3 (Bethesda). 2016 May 17;6(7):2073–9. doi: 10.1534/g3.116.030841 (PMC4938660; doi:10.1534/g3.116.030841)
Supplement: Supplemental Material [file supp_g3.116.030841_TableS4.pdf]

Table S4. Logistic regression analysis for PLG-tagging SNPs. P-values were corrected for age and gender. Odds ratios (OR) and 95% confidence intervals (CI) are provided on the minor allele.

dbSNP	Genotypes	Controls n (%)			Multiple sclerosis n (%)			p-value	OR (95% CI)
**rs9458005**	AA/AG/GG	476 (0.58)	297 (0.36)	51 (0.06)	1193 (0.60)	705 (0.35)	98 (0.05)	0.618	0.92 (0.78-1.09)
**rs2144723**	CC/CT/TT	231 (0.28)	409 (0.50)	182 (0.22)	557 (0.28)	990 (0.50)	448 (0.22)	0.274	1.01 (0.84-1.21)
**rs1830519**	AA/AG/GG	438 (0.53)	323 (0.39)	61 (0.07)	1120 (0.56)	742 (0.37)	135 (0.07)	0.154	0.89 (0.76-1.05)
**rs783147**	GG/GA/AA	244 (0.30)	394 (0.48)	184 (0.22)	599 (0.30)	1031 (0.52)	366 (0.18)	0.307	0.98 (0.82-1.18)
**rs783146**	CC/CG/GG	571 (0.69)	225 (0.27)	28 (0.03)	1420 (0.71)	528 (0.26)	46 (0.02)	0.265	0.91 (0.76-1.09)
**rs2295368**	GG/GA/AA	301 (0.37)	402 (0.49)	121 (0.15)	656 (0.33)	1007 (0.50)	333 (0.17)	0.256	1.18 (0.99-1.39)
**rs4252135**	GG/GT/TT	414 (0.50)	332 (0.40)	77 (0.09)	1008 (0.51)	820 (0.41)	163 (0.08)	0.719	0.99 (0.84-1.16)
**rs4252170**	TT/TC/CC	683 (0.83)	129 (0.16)	13 (0.02)	1682 (0.84)	298 (0.15)	14 (0.01)	0.789	0.89 (0.72-1.11)
**rs783176**	AA/AG/GG	556 (0.67)	228 (0.28)	41 (0.05)	1375 (0.69)	557 (0.28)	65 (0.03)	0.065	0.93 (0.79-1.11)
