# Supplementary material for: Analysis of Plasminogen Genetic Variants in Multiple Sclerosis Patients
Source: G3 (Bethesda). 2016 May 17;6(7):2073–9. doi: 10.1534/g3.116.030841 (PMC4938660; doi:10.1534/g3.116.030841)
Supplement: Supplemental Material [file supp_g3.116.030841_TableS5.pdf]

Table S5. PLG-coding variants identified in MS patients. Allele frequencies from the NHLBI GO Exome Sequencing Project (ESP), the 1000 Genomes Project (1000G), and The Exome Aggregation Consortium (ExAC) are provided. Chromosomal positions are provided in reference to NCBI Build 37.1. NR, not reported.

Chr	Position	Nucleotide change	Protein change	dbSNP rs/ss ID	Minor allele frequency		
					ESP	1000G	ExAC
6	161127501	A/G	p.K38E	rs73015965	0.004	0.003	0.003
6	161128812	G/A	p.R89K	rs143079629	0.008	0.003	0.007
6	161132146	C/T	p.N110N	rs4757	0.37	0.25	0.26
6	161134069	G/A	p.R153R	rs144153702	0.001	NR	0.001
6	161135876	A/G	p.T200A	rs149145958	0.001	0.001	0.001
6	161137779	T/C	p.C257C	rs14224	0.45	0.45	0.42
6	161137790	G/A	p.R261H	rs4252187	0.003	0.002	0.003
6	161139480	C/T	p.F314F	rs1130656	0.36	0.37	0.38
6	161139857	A/G	p.Q361Q	rs13231	0.26	0.16	0.22
6	161152107	G/A	p.R427R	rs149909079	0.004	0.001	0.003
6	161152155	C/T	p.S443S	ss1467426691	NR	NR	0.00001
6	161152206	T/A	p.S460R	rs116573785	0.01	0.02	0.006
6	161152240	G/A	p.D472N	rs4252125	0.26	0.16	0.22
6	161152257	C/T	p.S477S	rs4699	0.003	0.002	0.001
6	161152807	G/A	p.R490Q	rs140537724	0.002	0.001	0.001
6	161152819	C/T	p.A494V	rs4252128	0.009	0.01	0.008
6	161152837	C/T	p.T500M	rs140970354	0.0004	0.001	0.0002
6	161152858	C/T	p.A507V	rs372603134	0.0002	NR	0.0001
6	161152905	C/T	p.R523W	rs4252129	0.01	0.003	0.007
6	161159619	T/C	p.L618L	rs4252195	0.005	0.001	0.003
6	161162406	T/C	p.A694A	rs4252170	0.06	0.08	0.07
6	161173946	G/T	p.G762G	rs11060	NR	0.31	0.61
