# Supplementary material for: Analysis of Plasminogen Genetic Variants in Multiple Sclerosis Patients
Source: G3 (Bethesda). 2016 May 17;6(7):2073–9. doi: 10.1534/g3.116.030841 (PMC4938660; doi:10.1534/g3.116.030841)

Figure S2. Segregation analysis of PLG variants not observed in controls. Males are represented by squares and females by circles, with the proband indicated with an arrow head. Patients diagnosed with MS have black filled symbols. Heterozygote carriers (M) and wild-type (wt) genotypes are indicated. Inferred carriers are indicated with an asterisk.

PLG p. T200A (rs149145958)

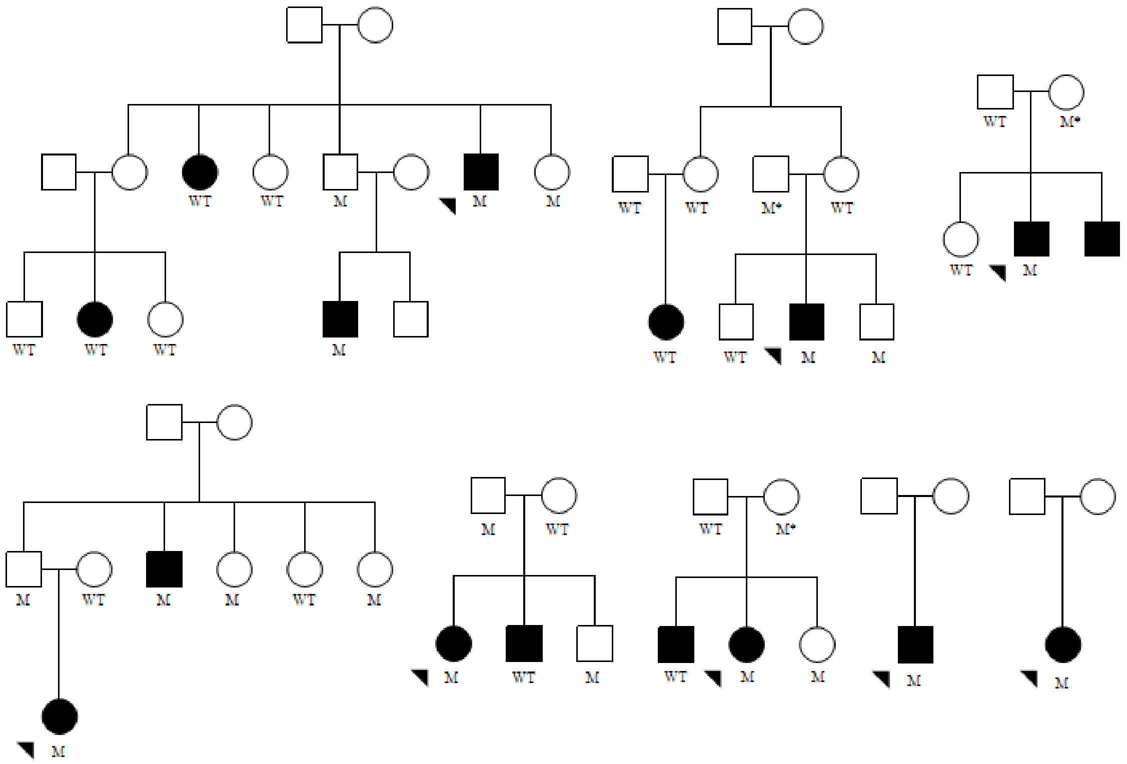

PLG p.T500M (rs140970354)

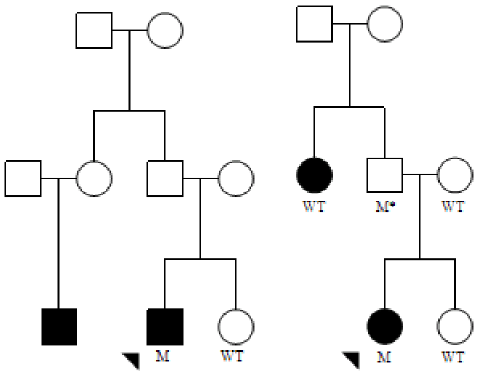

PLG p.A507V (rs372603134)

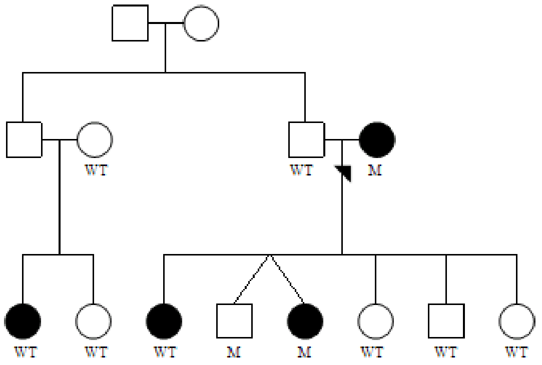

Supplement: Supplemental Material [file supp_g3.116.030841_FigureS2.pdf]
